# Hierarchical Scaling in Systems of Natural Cities

**DOI:** 10.3390/e20060432

**Published:** 2018-06-04

**Authors:** Yanguang Chen, Bin Jiang

**Affiliations:** 1 Department of Geography, College of Urban and Environmental Sciences, Peking University, Beijing 100871, China; 2 Faculty of Engineering and Sustainable Development, Division of GIScience, University of Gävle, SE-801 76 Gävle, Sweden

**Keywords:** allometry, hierarchy, scaling, fractals, entropy, natural cities

## Abstract

Hierarchies can be modeled by a set of exponential functions, from which we can derive a set of power laws indicative of scaling. The solution to a scaling relation equation is always a power law. The scaling laws are followed by many natural and social phenomena such as cities, earthquakes, and rivers. This paper reveals the power law behaviors in systems of natural cities by reconstructing the urban hierarchy with cascade structure. Cities of the U.S.A., Britain, France, and Germany are taken as examples to perform empirical analyses. The hierarchical scaling relations can be well fitted to the data points within the scaling ranges of the number, size and area of the natural cities. The size-number and area-number scaling exponents are close to 1, and the size-area allometric scaling exponent is slightly less than 1. The results show that natural cities follow hierarchical scaling laws very well. The principle of entropy maximization of urban evolution is then employed to explain the hierarchical scaling laws, and differences entropy maximizing processes are used to interpret the scaling exponents. This study is helpful for scientists to understand the power law behavior in the development of cities and systems of cities.

## 1. Introduction

Hierarchy is one of the basic characters of complex systems such as cities and networks of cities. A hierarchy can be mathematically described with a power law or a pair of exponential laws. In recent years, many scientists have been interested in the hierarchical structures of natural and social systems [1]. A fractal object is a self-similar hierarchy [2,3]. According to the ideas from fractal cities, a city or a system of cities can be treated as a hierarchy with a cascade structure [4,5]. A finding is that a self-similar hierarchy can be described with two or three exponential functions, from which it follows a set of power functions indicative of scaling [6,7]. This suggests that although the scaling in cities can be described with power laws, it can be understood through exponential laws. Scaling is a transformation that dilates (enlarges, increases) or contract (shrinks, diminishes) an object by a given scale factor. If the transformed result based on any scale factor bear the same structure with the original object, we will say that the process obeys scaling law [3,8]. The idea from scaling is very important to model scale-free phenomena. More and more scientists become aware of the importance of scaling analysis in urban studies [2,4,6,9,10,11,12,13,14]. Meanwhile, a number of puzzling issues arise from the research on scaling of cities [15,16]. Many questions are still pending and require much more studies before finding satisfying answers to them. Anyway, scaling laws often reveal the general principles underlying the structure of a physical problem [17]. Scaling analysis is an effective approach to urban spatio-temporal and hierarchical modeling. Scaling relations take on power laws, and a power law can be decomposed into two exponential laws based on hierarchical structure. Exponential laws can be derived by using the method of entropy maximizing [7], and this implies that the principle of entropy maximization can be utilized to interpret the power laws and thus explain the scaling behaviors of cities.

The hierarchical scaling laws are associated with many mathematical laws of cities. The models are found and reconstructed by Chen [6], who once explored the relationships between Zipf’s law [18], Christaller’s central place hierarchy [19], Beckmann’s city hierarchy model [20], Davis’ 2*^n^* rule [21], and Berry-Woldenberg’s analogy between rivers and central places [22]. Hierarchical scaling analysis can be employed to de-noise city rank-size distributions and reveal the regularity of urban evolution. This paper is devoted to revealing and describing the deep structure of systems of natural cities using the hierarchical scaling relations. We agree with Pumain [1] who once argued that the analysis of the hierarchical organization of complex systems such as cities can provide new insight for understanding systems’ evolution and emergence of order. The main contents of this paper are arranged as follows. In Section 2, to make it easier for readers to understand this work, the mathematical expressions of exponential laws and power laws for hierarchical structure are illuminated. In particular, the principle of entropy maximization is employed to explain the power law behaviors of natural cities. In Section 3, three hierarchical scaling laws are applied to the data sets of natural cities from the U.S.A., Britain, France, and Germany, and the results are illustrated with the ideas from hierarchical scaling. In Section 4, the main points are summarized, and the related questions are discussed. Finally, we reach the chief conclusions of this study.

## 2. Theoretical Models

### 2.1. Hierarchical Exponential Laws

The urban hierarchy with cascade structure can be described from two complementary angles of view. The longitudinal distributions can be described with exponential functions, and the latitudinal relationships can be described with power functions [7,23]. Considering a geographical region with *n* cities, we can organize the cities into a hierarchy comprising *M* classes according to the generalized 2*^n^* rule [7,24]. Based on the top-down order, the cascade structure of the urban hierarchy can be modeled by a set of exponential functions such as:

(1) $$N_{m}=N_{1}r_{n}^{m-1},$$

(2) $$S_{m}=S_{1}r_{s}^{1-m},$$

(3) $$A_{m}=A_{1}r_{a}^{1-m},$$

where *m* refers to the order number of city class (*m* = 1, 2, …, *M*), *N_m_* denotes the number of cities of order *m*, correspondingly, *S_m_* and *A_m_* represent the average population size and average urban area at the *m*th class. The parameters are as below: *N*_1_ denotes the number of the top-class cities, *S*_1_ and *A*_1_ are the mean size and mean area of the first-class cities, *r*_n_ = *N_m_*_+1_/*N_m_* refers to the interclass *number ratio* of cities, *r*_s_ = *S_m_*/*S_m_*_+1_ to the city *size ratio*, and *r*_a_ = *A_m_*/*A_m_*_+1_ to the urban *area ratio*. Generally speaking, *N*_1_ = 1, but for the three-parameter Zipf’s distribution, *N*_1_ > 1 [25]. Equations (1)–(3) compose the mathematical expressions of the generalized 2*^n^* principle [7], which is based on Beckmann-Davis models [20,21]. Davis find the 2*^n^* rule that says that if the cities in a geographical region are organized into a hierarchy by means of a fixed size ratio *r*_s_ = 2, the number ratio of the cities at different levels will be 2, that is, *r*_n_ = 2 [21]. This principle can be generalized to the following statement: if the cities in a region are organized into a hierarchy with cascade structure by means of a fixed number ratio *r*_n_ = 2, the size and area ratio of the cities at different levels will range from 1 to 3, respectively, that is, 1 < *r*_s_ < 3, 1 < *r*_a_ < 3. The is called generalized 2*^n^* rule of hierarchies of cities [7,24].

Among this set of exponential functions, the first equation represents the *number law*, the second equation represents the *size law*, and the third equation represents the *area law* of urban hierarchies [6]. The three exponential laws can be derived by using the method of entropy maximizing [7]. For a self-similar hierarchy, if *r*_n_ = 2 as given, then it will follow that *r*_s_→2, and if *r*_s_ = 2 as given, then it will follow that *r*_n_→2, where the arrow denotes “approach”. If *r*_n_ = *r*_s_ = 2, the generalized 2*^n^* principle will return to Davis’ standard 2*^n^* principle. In this instance, we will have *T_m_* = *N_m_S_m_* = *N*_1_*S*_1_ = *S*_1_, where *T_m_* denotes the total population at the *m*th level. This suggests a property of hierarchical conservation of size distributions, which is consistent with the standard 2*^n^* rule of cities. If a hierarchy of cities complies with Davis’ 2*^n^* rule, the total population size at each level of the hierarchy is theoretically a constant.

A set of Zipf’s models is hidden behind the three exponential laws. This suggests that Zipf’s distributions can act as an indication of the self-similar hierarchy. From Equations (1)–(3) we can derive Zipf’s laws of population size distribution and area size distribution. The city size can be measured by both population quantity and urbanized area. The former is termed population size, and the latter is called area size of a city. Where population size distribution is concerned, three types of Zipf’s models can be derived [26]. If *r*_n_ = *r*_s_, we can derive a one-parameter Zipf’s model, *S*(*k*) = *S*_1_/*k*, where *k* refers to rank, and *S*_1_ is a parameter indicative of the largest city [27,28]. The one-parameter Zipf’s model is termed pure Zipf’s law in literature [29]. If *r*_n_ ≠ *r*_s_, we can derive a two-parameter Zipf’s model, *S*(*k*) = *S*_1_/*k^q^*, where *q* is the second parameter indicating scaling exponent. If *r*_n_ ≠ *r*_s_ and the largest city cannot influence the whole geographical region, we can derive a three-parameter Zipf’s model, *S*(*k*) = *C*/(*k* + *h*)*^q^*, where *h* is the third parameter indicating translational factor, and *C* denotes proportionality coefficient [26]. Where there are rank-size distributions of cities which follows Zipf’s law, there is a hierarchy of cities with cascade structure, and *vice versa* [6,7].

### 2.2. Hierarchical Power Laws

The relationships between exponential laws and power laws suggest the relationships between simplicity and complexity. Especially, these relationships suggest the links between characteristic scales and scaling. Exponential laws indicate the conventional growth, distributions, and processes with characteristic scales, while power laws indicate the allometric growth, fractals, and patterns without characteristic scales. The former suggests simplicity, and the latter suggests complexity. The exponential laws and power laws can be integrated into the same framework with the hierarchical scaling concept. The hierarchical scaling in cities performs power law behaviors and can be expressed with the three power functions [7,30]. From the above exponential laws based on longitudinal distributions, it follows a set of power-law equations for latitudinal relationships as follows [7]:

(4) $$N_{m}=\mu S_{m}^{-D},$$

(5) $$N_{m}=\eta A_{m}^{-d},$$

(6) $$A_{m}=aS_{m}^{b},$$

where the parameters can be expressed as *μ* = *N*_1_*P*_1_*^D^*, *D* = ln*r*_n_/ln*r*_s_, *η* = *N*_1_*A*_1_*^d^*, *d* = ln*r*_n_/ln*r*_a_, *a* = *A*_1_*P*_1_^−*b*^, and *b* = ln*r*_a_/ln*r*_s_. Among these parameters, *D* denotes the fractal dimension of city population size distributions, *d* denotes the fractal dimension of urban area size distributions, and *b* is the allometric scaling exponent of urban hierarchy. In fact, *b* is the ratio of the fractal dimension *D* to the dimension *d*, that is, *b* = *D*/*d* = (ln*r*_n_/ln*r*_s_)/(ln*r*_n_/ln*r*_a_) = ln*r*_a_/ln*r*_s_. Apparently, from Equations (1) and (2), we can derive Equation (4); from Equations (1) and (3), we can derive Equation (5); from Equations (2) and (3), or from Equations (4) and (5), we can derive Equation (6). This implies that, for the cascade structure of a hierarchy of cities, exponential laws and power laws represent two different sides of the same coin. The exponential laws can be directly derived from the principle of entropy maximization, and thus entropy maximization can be employed to indirectly explain the power laws of cities.

**Figure 1:**
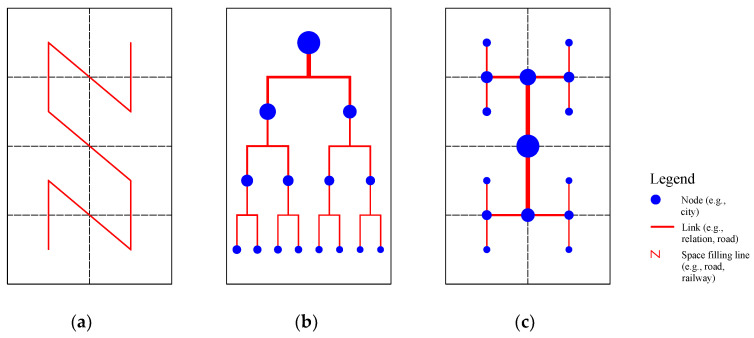
Spatial recursive subdivision, hierarchy, and network structure of cities. Note: The rank-size distribution of cities can be organized into a self-similar hierarchy, which corresponds to a cascade network. The network structure is based on strict recursive subdivision of geographical space [4,32]. (**a**) Spatial subdivision; (**b**) Hierarchy; (**c**) Network.

The above-shown power laws represent three typical aspects of scaling behaviour of cities. Equation (4) suggests the *size-number scaling* in a hierarchy of cities. It is equivalent to the Pareto law of population size distribution, and *D* is the fractal dimension of urban hierarchies measured with city size such as population [26]. Equation (5) suggests the *area-number scaling* of cities. It is equivalent to the Pareto law of city-area distribution, and *d* can be regarded as the fractal dimension of urban hierarchies measured with urbanized area [30]. Equation (6) suggests the hierarchical allometric scaling relation between urban area and size, and *b* is the allometric scaling exponent of urban hierarchy [31]. The inverse functions of Equations (4) and (5) are equivalent to the Zipf’s laws of population size distribution and area size distribution. This once again implies that Zipf’s distribution is just a signature of hierarchical scaling. In scientific research, one of difficult problems of mathematical modeling rests with spatial dimension [25]. Hierarchy and network represent two different sides of the same coin [4]. Network structure is associated with spatial recursive subdivision [32]. By hierarchical scaling analysis, we are able to find new way of modeling spatial distribution and network organization (Figure 1).

### 2.3. Entropy Maximization and Power Laws

The power law behaviors of hierarchical scaling in city development can be explained by the principle of entropy maximization. In urban hierarchies, a power law is based on two exponential laws, and the relationships between the power laws and exponential laws can be revealed by the self-similar hierarchy. In fact, exponential distributions can be directly derived by using entropy-maximizing methods [7,33,34,35,36,37], and a power law can be directly derived from a pair of exponential laws [6,7,35,37]. Thus, the power laws can be indirectly derived from the principle of entropy maximization of urban evolution. Exponential functions bear the property of mirror symmetry, that is, changing the direction of the independent variable will not change the functional structure, but the exponential function will change to negative exponential function and vice versa [6]. Based on this property, Equations (1)–(3) can be derived by means of the entropy maximization principle [7]. As indicated above, city size can be measured by both urban population and urbanized area. The three exponential models represent three different but related processes of entropy maximization of city development (Table 1). Equation (1) is based on the entropy maximization process of the frequency distribution of city numbers, Equation (2) is based on the entropy maximization of the size distribution of city population, and Equation (3) is based on the entropy maximization of the size distribution of urbanized area.

**Figure 2:**
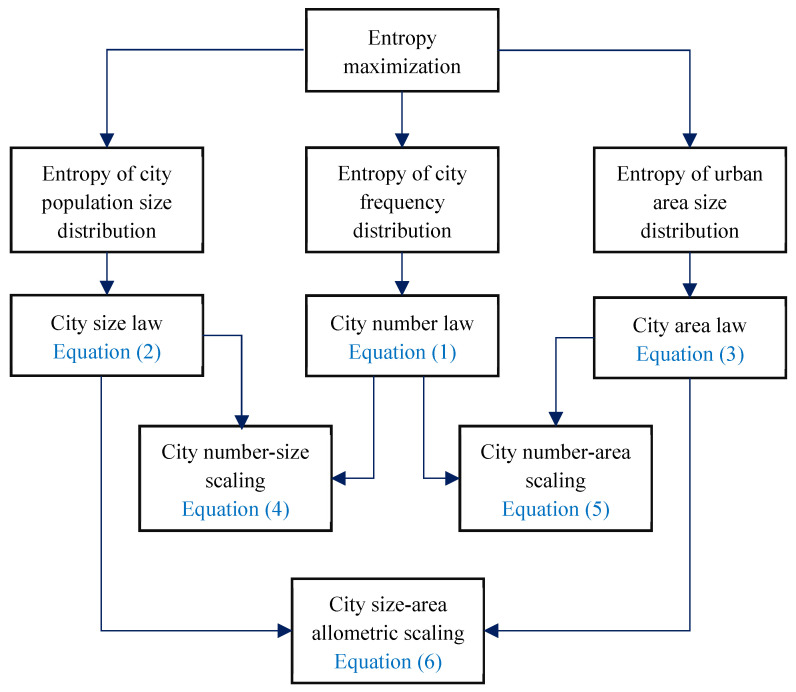
The relationships between the principle of entropy maximization and the hierarchical scaling laws of cities. Note: Using the method of entropy maximizing, we can derive three exponential laws on the longitudinal distributions of urban hierarchies, but we cannot derive the three power laws for the latitudinal relationships of cities. By the hierarchical structure, we can derive the power laws indirectly with the entropy maximizing method through the exponential laws of cities.

Now, the principle of entropy maximization can be employed to explain the emergence of power law behaviors of cities. In the hierarchy with cascade structure, power laws and exponential laws are compatible with each other. A pairs of exponential laws indicative of longitudinal distributions support a power law reflecting the latitudinal relation between two measurements. In this work, the power laws, Equations (4)–(6), are derivable from the exponential laws, Equations (1)–(3). This suggests that a power law is based on two dual processes of entropy maximization (Figure 2). Concretely speaking, the city size-number scaling, Equation (4), is based on the entropy maximization process of city population size distribution and that of city frequency distribution; the city area-number scaling, Equation (5), is based on the entropy maximization process of urban area size distribution and that of city frequency distribution; and the allometric scaling relation between city population and urbanized area, Equation (6), is based on the entropy maximization process of city population size distribution and that of urban area size distribution.

Moreover, the principle of entropy maximization can also be utilized to interpret the scaling exponent values of power laws of cities. If the two dual processes of entropy maximization are of synchronization and in a state of balance, the scaling exponent will be close to 1, or else, the exponent value will departure from 1 (greater than or less than 1). Generally speaking: (1) if the entropy maximization process of the frequency distribution of cities numbers and that of the city population size distribution keep in step with each other and fall in the state of balance, the scaling exponent *D* in Equation (4) approaches to 1, i.e., *D*→1, otherwise, *D* > 1 or *D* < 1; (2) if the entropy maximization process of the frequency distribution of cities numbers and that of the urban area size distribution keep in step with each other and fall in the state of balance, the scaling exponent *d* in Equation (5) approaches to 1, i.e., *d*→1, otherwise, *d* > 1 or *d* < 1; (3) if the entropy maximization process of the city population size distribution and that of the urban area size distribution keep in step with one another and fall in the state of balance, the scaling exponent *b* in Equation (6) approaches to 1, i.e., *b*→1, otherwise, *b* > 1 or *b* < 1. Using the power law relations and scaling exponents based on the entropy maximization, we can make evaluation on city development in a geographical region.

## 3. Empirical Analysis

### 3.1. Study Area, Data, and Methods

The validity and rationality of the mathematical models can be verified and evaluated through empirical observation data. In fact, the success of natural sciences just rests heavily with their great emphasis on the role of interplay between quantifiable data and models [16]. Four systems of cities in Europe and the U.S.A. can be employed to testify the hierarchical scaling laws and the related models about cities. Jiang and his coworkers [38,39] proposed a concept of *natural city* and developed a new approach to measuring objective city sizes using street nodes or blocks. In urban geography, a city can be defined as a large settlement that has some kind of service functions to the surrounding areas. However, a natural city is the human settlement based on landscape rather than service functions. Natural cities proposed by Jiang and his co-workers [38,39] can be understood by two basic principles of geography: one is the man-land relations, and the other, the distance-decay effect. For urban form and growth, the man-land relations can be expressed by the allometric scaling relations between urban population and land [6]. Human activities and city population size can be reflected on the urban land use. On the other hand, human population activity density of an urban region decreases from center to periphery with distance [4,6]. Thus, according to the distance decay law, we can identify the boundary lines of urban population activities by using some methods. The urban boundary can be termed “urban envelope” [4,40]. In terms of the man-land allometric relations, an urban envelope represents an urban place and reflects the city size. So, each envelope can be treated as a boundary of natural city. The key rests with how to identify urban boundaries. Based on remote sensing images or digital maps, at least three approaches have been developed to determine urban envelopes for cities. The first is the city clustering algorithm (CCA) proposed by Rozenfeld and his co-workers [41,42], the second is the method of clustering street nodes/blocks advanced by Jiang and Jia [38], and the third is the fractal-based method presented by Tannier and his co-workers [43]. In this paper, the natural cities are extracted by means of the method proposed by Jiang and Jia [38]. Using this approach, we can obtain large datasets of natural cities. Compared with the cities in the usual sense, the rank-size distributions of natural cities are very robust and bear a longer scaling range. Recent years, Jiang and his co-workers developed new approach such as head-tail index to identify natural cities [44,45].

Urban block is an ordinary concept, and the street nodes are defined as street intersections and ends. Using an identification algorithm of urban boundary, Jiang’s research group was able to delineate boundaries of natural cities and yield city areal extents. Thus urban area can be determined by a city’s areal extent containing a large number of street blocks or nodes. The number of street nodes is significantly correlated with the population size of cities. The city data are extracted from massive volunteered geographic information OpenStreetMap databases through some data-intensive computing processes, and four datasets on the cities of the U.S.A., Britain (UK), France, and Germany are available. The process of identifying natural cities is actually an approach of spatial search, and the number of cities is automatically determined through spatial search technique. By the same technical criterion of spatial search, the numbers of natural cities extracted from different countries may be very different. The reason lies in the different geographical conditions, which result in great differences in the spatial patterns of urban development. In Britain and France, natural cities have better correspondence with the usual cities, while in Germany, natural cities are significantly different from traditional ones.

The empirical analysis can start from investigating Zipf’s distribution, which, as pointed out above, can be regarded as a signature of the hierarchies with cascade structure. If cities in a region follow Zipf’s law, they can be organized into a self-similar hierarchy [7,30]. It has been shown that the cities in the four countries follow Zipf’s law [38,39]. Applying the generalized 2*^n^* rule to the above-mentioned datasets, we can create four self-similar hierarchies of European and U.S. cities. Suppose that these systems of cities follow the pure Zipf’s law. Then the cities in each country can be reorganized into a hierarchy with cascade structure. The Zipf’s law cannot be directly derived by using the method of entropy maximizing, but the hierarchical scaling laws can be derived by means of this approach. Curry once tried to derive Zipf’s law using the idea from entropy maximization [33], but his result is actually a three-parameter exponential function rather than a power function [7]. Table 2 is presented for understanding the operational process of hierarchical reconstruction (two Supplementary Material files are provided to show how to process the data and estimate the scaling exponents, see Files S1 and S2).

Several algorithms can be adopted to evaluate the scaling exponents. The most common ones include the least squares method (LSM) [37], maximum likelihood method (MLM) [46,47], and major axis method (MAM) [26,48]. Recent years, the MLM is often used to identify power-law distributions, and it is treated as the most available approach to estimating power exponents. In fact, the power-law relations of this work are based on exponential functions, and are converted into logarithmic linear relations. It was demonstrated that if the observations come from an exponential family and mild conditions are satisfied, the least-squares estimates are identical to the maximum-likelihood estimates [49]. What is more, if the errors of a linear model belong to the normal distribution, the least squares estimators are also identical to the maximum likelihood estimators. All in all, the function of an algorithm is to estimate the parameter values of a mathematical model rather than judge the form of a model’s expression. Any algorithm has its advantages and disadvantages, sphere of application, and applicative conditions. The precondition of applying the MLM to observational data is that the variables satisfy the joint normal distribution. Unfortunately, for human systems such as cities, the observational data do not always meet the joint normal distribution. In this case, the LSM is an advisable approach to estimating power exponent values [23,37]. The models’ parameters are evaluated by using the least squares calculations.

### 3.2. Results and Findings

The systems of cities in the U.S.A., U.K., France, and Germany can be well described with hierarchical scaling equations. In light of the generalized 2*^n^* principle expressed by Equations (1) and (2), we can organize the cities in each country into a hierarchy with cascade structure. The city number in the *m*th level is *N_m_* = 1, 2, 4, …, 2*^m^*^−1^, … The numbers of levels in the urban hierarchies in the four countries are 15, 11, 11, and 13, respectively. The last levels are lame-duck classes because that city numbers are not big enough. Based on the hierarchical structure, we can calculate the average city size *P_m_* and the corresponding average urban area *A_m_* at each level (Table 3). The city numbers in different classes are designed according to the 2*^n^* rule and satisfy Equation (1). It is easy to testify that city size *P_m_* and urban area *A_m_* follow exponential distribution and meet Equations (2) and (3), respectively, but the lame-duck classes are two outliers due to lack of adequate cities. Strictly speaking, the first class is usually an outlier because the largest city is often an exception [7]. In fact, a mathematical law always becomes ineffective when the scale of measurement is too large or too small.

**Figure 3:**
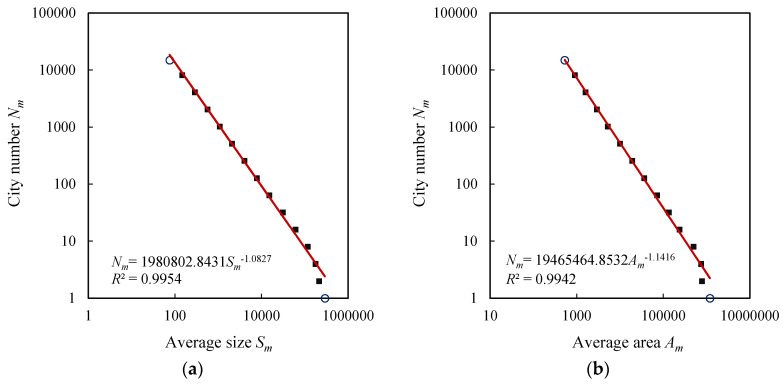
The hierarchical scaling relationships between size (block/street node quantity) and area (physical extent) of U.S. cities. Note: The small circles represent top classes and the lame-duck classes, respectively. Removing the first and last classes yields a scaling range. The slopes based on the scaling ranges indicate the fractal parameters of city size and area distributions. The ratio of the size dimension *D* to the area dimension *d* is close to the allometric scaling exponent *b*, i.e., *b* ≈ *D*/*d*. Similarly hereinafter. (**a**) City size; (**b**) Urban area.

**Figure 4:**
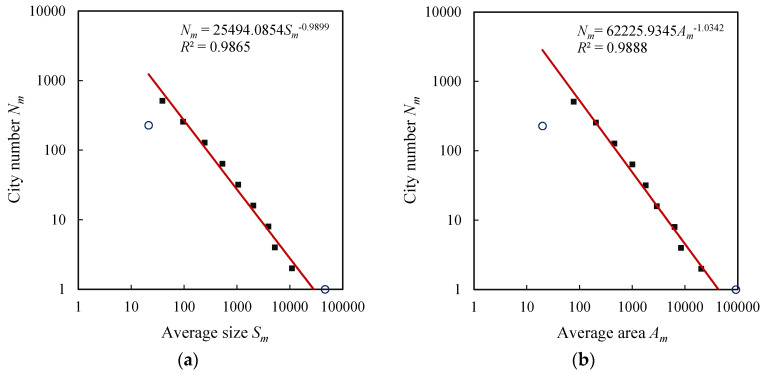
The hierarchical scaling relationships between size (block/street node quantity) and area (physical extent) of British cities. (**a**) City size; (**b**) Urban area.

**Figure 5:**
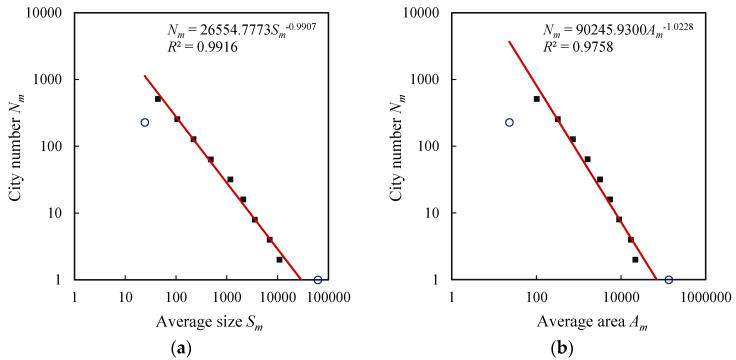
The hierarchical scaling relationships between size (block/street node quantity) and area (physical extent) of French cities. (**a**) City size; (**b**) Urban area.

**Figure 6:**
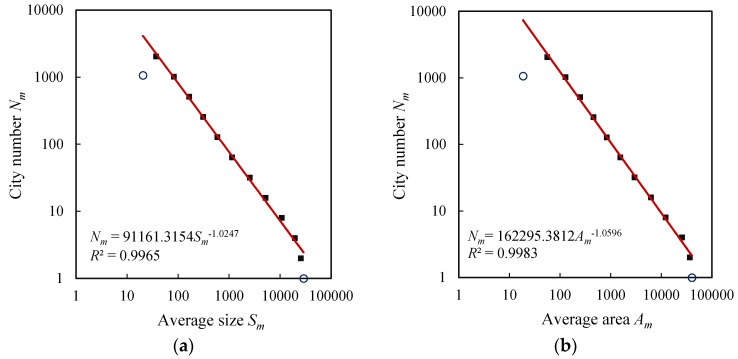
The hierarchical scaling relationships between size (block/street node quantity) and area (physical extent) of German cities. (**a**) City size; (**b**) Urban area.

The exponential distributions of city size and urban area result in the power-law relations between city number, size, and area. The exceptional values in the exponential laws often manifest themselves on the log-log plots for power laws. In fact, if the scale is too large or too small, a power-law relation always breaks down [6,50]. Thus the extreme classes always form exceptional points, and there exists a scaling range between the two extremes. For U.S. cities, the last class of cities is out of trend lines and forms outliers, but the first class of cities is normal (Figure 3). For the British, French, and German cities, both the first and last classes are exceptional values (Figure 4, Figure 5 and Figure 6). For comparability, the first class of U.S. hierarchy of cities is treated as an outlier, which does not influence the results and conclusions significantly. Removing the first and last data points as outliers yields the ranges for the scaling relations between city number and city size or urban area. All the data points within the scaling range follow power law and take on double logarithmic linear relationships. In particular, the influence of primate distribution of city sizes on the hierarchical scaling patterns is weak. In urban geography, city size distributions are divided into two different groups: rank-size distribution and primate distribution [6]. In short, without considering the first and last classes, the relation between city size and number can be described with Equation (4), and the relation between urban area and city number can be described with Equation (5). Fitting Equations (4) and (5) to the datasets in Table 3, we can evaluate the parameters by the least squares calculation. The scaling exponent values are close to 1, and the *d* value (area exponent) is slightly greater than the *D* values (size exponent). The ratio of *D* to *d* can be termed fractal dimension quotient of urban hierarchies. As indicated above, if *D* approaches 1, the total “population” of the *m*th level approaches a constant *S*_1_. Despite the fact that there are always many smaller cities than larger ones [29,38,45], the product of average size and city number at each class seems to be invariable. This reminds us of the work of Auerbach, who asserted the product of the population size of city class *i*, *P_i_*, and the rank of class *i* within all classes when ordered by population size, *R_i_*, approaches a constant *K*, i.e., *P_i_R_i_* = *K* [51]. The difference rests with that Auerbach’s finding is a special case of Zipf’s formula, which represents the restrictive rank-size rule rather than the hierarchy with cascade structure. In our context, the total size in the *m*th level of the self-similar hierarchy, *T_m_* = *N_m_S_m_*, approaches to a constant, i.e., *T_m_*→*constant*, which suggests a conversation law. The conversation law implies some type of symmetry [6,52]. In this study, the conversation is associated with hierarchical scaling symmetry.

Since Zipf’s law is a signature of the self-similar hierarchy of cities, two distributions related to the rank-size distributions should be discussed here. First, the relationship between Zipf’s distribution and the lognormal distribution. Where city-size distributions are concerned, if we do not identify the scaling range, the rank-size relation often satisfy a lognormal distribution rather than a power-law distribution; however, if the scaling range is taken into consideration, the power-law relation is always clearer than the lognormal relation. In order to reveal the power-law relations of urban hierarchies, the data points at the two extremes should be removed as outlier. Second, the rank-size distribution and the primate distribution. Both the city size distributions of Britain and France are regarded as primate distribution. However, according to Figure 4 and Figure 5, the primate distribution seems not to represent an independent type. The large cities in Britain and France take on the character of primate distribution because London and Paris are two global cities [6]. The primate distribution has impact on the log-log relations between city number, size, and urban area. However, this influence is not significant to the hierarchical scaling relations based on large datasets. This seems to suggest that, compared with the rank-size law, the primate law represents a local rule rather than a global principle of city size distributions.

**Figure 7:**
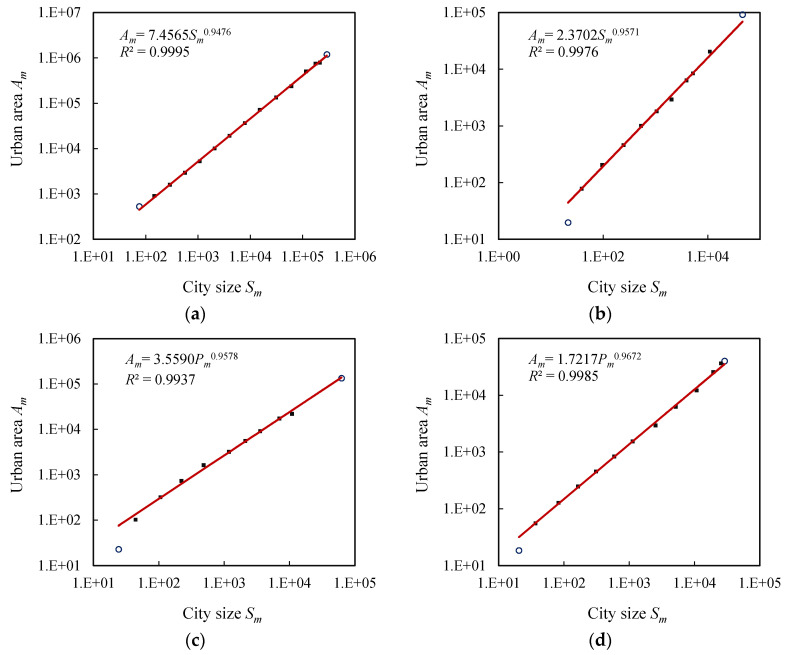
The hierarchical allometric scaling patterns of four systems of natural cities (U.S.A., Britain, France, and Germany). Note: The small circles represent the top class indicative of the largest city and the bottom class indicative of the small towns. The trend lines are based on the data points within the scaling ranges. (**a**) U.S.A.; (**b**) Britain; (**c**) France; (**d**) Germany.

The relationships between city number and city size or urban area are a pair of fractal dimension relations, from which it follows an allometric scaling relation between city size and urban area. Using the data displayed in Table 3, we can estimate the allometric scaling exponent values. Corresponding to the exponential models and fractal models above mentioned, the first and last classes are treated as outliers so that the allometric parameters and fractal parameters are more comparable with one another. The allometric scaling of the hierarchies of cities in the four European and American countries is clear and significantly convincing. For U.S. cities, all the data points follow the allometric scaling law; for the cities of the U.K., France, and Germany, the last levels, i.e., the lame-duck classes, are exceptional points (Figure 7). The main results are shown in Table 4, in which we can see the way and effect of data processing.

The four study areas, U.S.A., U.K., France, and Germany, are all developed countries, and the levels of urbanization are near their respective capacity values, i.e., the upper limit values. The allometric scaling properties of these urban hierarchies are as below: First, the allometric scaling exponent is close to but less than 1. This suggests that the relative growth rate of city size is slightly less than that of urban area. When a city is small, its population density is low, the per capita land use quantity is large, and the city expands fast in the two-dimensional space. With the growth of the city, the population distribution is becoming more and more concentrated, and the urban buildings begin to develop to the higher level, thus the per capita land consumption become smaller and smaller, and intensification of urban land use emerges. As a result, the allometric scaling exponent *b* ≤ 1. Second, the allometric scaling exponent is equivalent to the fractal dimension quotient. In theory, the allometric exponent is the ratio of the fractal dimension of urban population size distribution to that of urban area size distribution. Where empirical analysis is concerned, the allometric exponent is close to the fractal dimension ratio. Generally speaking, for the developing systems of cities, the fractal dimension of population size distribution is significantly less than that of area size distribution. The allometric scaling exponent values come between 2/3 and 1, and always approach to 0.85 [6,31,53]. However, for the developed cities, population growth and land use expansion reach the final equilibrium, and the difference between the two types of size distribution dimension is not significant. Therefore, the allometric scaling exponent is close to 1. Otherwise, a system will lose its balance [54]. The state of maximizing entropy balance indicates the suitablest scaling exponent value, for example, the Zipf’s exponent is *q* = 1 [7,27,28], and the urban area-population allometric scaling exponent is *b* = 1 or 0.85 [31,53]. If the two maximum entropy processes are seriously misaligned, the scaling exponent values will be abnormal. For instance, the Zipf’s exponent *q* must fall between 1/2 and 2, i.e., 1/2≤ *q* ≤ 2 [55], and the allometric scaling exponent exponent *b* must come between 2/3 and 1, i.e., 2/3 ≤ *b* ≤ 1 [31], or else, it suggests that the state of entropy balance of city size distribution and city frequency distribution is seriously damaged. According to the calculation results, for the natural cities in the developed countries, the three entropy maximization processes are approximately in step with each other and fall in the state of balance.

## 4. Discussion

The applications and functions of the self-similar hierarchy lie mainly in the following four aspects. First, it can be employed to reveal the physical foundations of power law behaviors of cities using the principle of entropy maximization [7]. Second, it can be used to integrated various related theories and models of cities such as central place theory, the rank-size rule, the 2*^n^* rule, gravity model, and so on into a logic framework [6,7,26,30]. Third, it can be utilized to make a bridge between temporal analysis, network analysis, and spatial analysis of cities [37]. Fourth, it can be used to bring to light the similarities and differences between the mathematical laws of human systems (e.g., cities) and those of natural systems (e.g., rivers) [6,31]. The empirical analysis shows that the natural cities of the three European and one North American countries follow hierarchical scaling laws. Taking scaling ranges into account, we can fit the size-number scaling and area-number scaling relations into the observational data very well. The two scaling relations are equivalent to the Zipf’s law of distributions of urban population and area [7]. The size-number scaling analysis gives the fractal dimension of population-size distribution *D*, and the area-number scaling analysis yields the fractal dimension of area-size distribution *d*. From the above-mentioned scaling relations, we can derive the size-area allometric scaling relation, and the allometric exponent *b* is equal to the ratio of *D* to *d*. All these results support the judgment that the evolution of natural cities conforms to the principle of entropy maximization. Entropy maximization means an optimal and coordinated relationships between the efficiency of the whole and the equity among individuals in a self-organized system [7,37]. In a sense, it is the competition and coordination between equality and the efficiency that lead to power law distribution of urban systems.

Hierarchical allometry is one of urban allometric scaling relations. Allometric scaling includes longitudinal allometry (temporal allometry), transversal allometry [56], and spatial allometry [6], and transversal allometry includes cross-sectional allometry based on rank-size distribution and hierarchical allometry based on cascade structure. The longitudinal allometry is based on exponential growth, or logistic growth [57], while the transversal allometry is based on rank-size distribution, exponential distribution, or hierarchical power-law distribution [6,58]. The hierarchical allometry is equivalent in mathematics to the cross-sectional allometry, and form a connection between transversal allometry and longitudinal allometry (Table 5). A hierarchy makes a link between spatial disaggregation and network structure [4]. In fact, the cities of different sizes at a hierarchy corresponds to the cities at different phases of development [4]. By researching allometric scaling in hierarchies of cities, we will be able to find the inherent correlations between spatial patterns, temporal processes, and dynamic mechanisms of urban evolution. The allometric scaling has been applied to urban studies based on census data and statistical data [7,23,30,31,57]. However, the observational data of natural cities provide better evidences for the hierarchical allometric scaling laws. This comparison is based on statistical analysis instead of city concepts. The natural city is not always consistent with the usual city. The comparison in the sense of statistics is based on the degrees of freedom and the corresponding confidence levels (or significance levels). For different sampling results, the number of elements (e.g., cities) are different, and thus the degrees of freedom are different. However, the different degrees of freedoms can be converted into comparable confidence levels (e.g., 95% or 99%).

Allometric scaling indicates a power-law relation, which suggests a proportional relation between two measures. Therefore, allometry is involved with two concepts of modern mathematical modeling. One is spatial dimension, and the other is scaling range. Spatial dimension is one of the conundrums in mathematical description. A measure is proportional to another measure, if and only if the two measures bear the same spatial dimension. So a length is in proportion to the square root of an area, or to the cubic root of a volume. This principle has long been discovered by the ancient Greeks. In this sense, the allometric scaling exponent of size-area indicates the ratio of two spatial dimension values such as *b* = *D*/*d* = *D*_a_/*D*_s_, where *D* and *d* refer to the fractal dimensions of population and area size distributions, and *D*_s_ and *D*_a_ denote the fractal dimension of the spatial distributions of city population size and land use form [58]. In fact, *D* and *d* proved to be paradimension according to the relationships between Zipf’s law and hierarchical scaling law [30]. The concept of paradimension was sublated by Mandelbrot [3], but it is useful in the studies on fractal cities. Scaling range indicates an effective range for scale-free analysis of cities. The hierarchical allometry is based on Zipf’s distributions. The largest city and the very small towns may violate Zipf’s law and take on outliers on a double logarithmic plot. If the largest city (e.g., London) is a world city, and the area of its country (Great Britain) is not large, then the sphere of influence of the largest city will go far beyond the national area. As a result, the largest city becomes an outlier and the primate distribution will replace Zipf’s distribution of cities [6]. Meanwhile, the small towns may form outliers and go beyond the scaling range in a log-log plot due to undergrowth of city sizes [7,30]. In China, improper government intervention in urbanization often gives rise to urban structure abnormalities, which takes on outliers in datasets [37]. Sometimes, small cities or towns are developed in relative size, but the city number does not reach 2*^M^*, where *M* is a positive integer (See Table 2). Thus the bottom level of urban hierarchy forms a lame-duck class [21].

The merits of this study rest with data quality, dataset size, and mathematical models. On the one hand, all the observational data are based on the concept of natural cities and bear high quality. On the other, the size of datasets are very large compared with the traditional sample sizes for the rank-size analyses. Compared with the studies on urban hierarchies and rank-size distributions based on census data or statistical data, the datasets of natural cities are more suitable for hierarchical scaling analysis of cities. What is more, the models have performance of anti-disturbance of random noises. The main drawbacks of the work lie in two aspects. First, the city size is measured by numbers of block or traffic nodes rather than urban population. A city is a human settlement, and population size belongs to the first order dynamic models of cities [59]. Two central variables can be employed to research spatial dynamics of urban development: population and wealth [60]. If the relation between urban population and block/node number is linear, the number of blocks or traffic nodes can be used to replace urban population, otherwise, the real relation should be revealed. Second, the temporal dimension does not be considered. Only one year datasets are available, and we cannot examine the dynamic change of hierarchies of natural cities. Despite these shortcomings, the contribution of the paper is clear: we use four big datasets of high quality to verify the hierarchical scaling laws from urban angle of view, and the results can be explained by the principle of entropy maximization.

## 5. Conclusions

In this paper, we investigate the systems of natural cities in three European and one North American counties. Two measures are employed to reflect city size, one is the number of blocks, and the other is number of streets nodes. Different urban systems based on different size measurements lead to the same direction: all these systems of cities can be organized into hierarchies with cascade structure. The self-similar hierarchy can be described with a set of exponential laws based on longitudinal direction distributions: number law, population size law, and urban area law. The three exponential Equations can be equivalently transformed into a set of power functions for latitudinal (transversal) direction relations, the first one reflect the size-number scaling relation, the second one reflect the area-number scaling relation, and third one reflect the size-area allometric scaling relation. The self-similar hierarchy indicates a kind of deep structure of systems of cities and latent spatial order in urban evolution, which can be understood from the perspective of entropy maximization. The contribution of this work to the studies on scaling of cities rests with the following aspects. First, the principle of entropy maximization is employed to explain the power law behaviors of urban hierarchies from new angles of view. Three processes of entropy maximizing are used to interpret the emergence of power laws and the related scaling exponents. Second, a series of allometric scaling models are divided into three groups and put in order in a logic framework. The framework includes longitudinal allometry, crosssectional allometry, and hierarchical allometry. Third, an entire case study on hierarchies of natural cities are presented. The case may be helpful for readers to understand power law behaviors in hierarchies from a new angle of view.

The main conclusions of this study can be drawn as follows. First, the natural cities provide a new way of understanding the hierarchical scaling laws, which can be represented by a set of power functions. The three hierarchical models can be well fitted to the datasets of natural city size and urban area of the U.S.A., Britain, France, and Germany by taking the scaling range into consideration. In a sense, the natural city is a concept of cities based on geographical landscape, which differs from the traditional city concept based on human administration. Compared with the census data or statistic data of common cities, the observational data of natural cities show better effect of hierarchical scaling analysis. Moreover, the allometric scaling relation comes from a pair of rank-size scaling relations. In theory, the allometric scaling exponent is equal to the ratio of the fractal dimension of population size distribution to that of area size distribution; in practice, the allometric exponent is very close to the quotient of the two fractal dimension values of size distributions (esp. Britain and Germany). Second, the principle of entropy maximization can be employed to explain the emergence of power law behaviors in hierarchies of natural cities. A power law is based on a pair of exponential laws on city number and sizes. An exponential law can be derived by means of the method of entropy maximizing. Thus a power law is determined by two dual entropy maximization processes: the entropy maximization of city frequency distribution and that of city size distribution. An urban hierarchy is involved two types of entropy maximization: the frequency distribution of cities and size distributions of urban population and urbanized area. The fractal models are controlled by an entropy maximization process of frequency distribution and that of size distribution, while an allometric scaling relation is dominated by two entropy maximization processes of size distributions such as population size distribution and area size distribution. Entropy maximization can explain the power law behaviors of traditional city size distribution, but this principle seems to be more suitable for explaining the evolution and power law emergence of natural cities. What is more, the entropy maximization principle can also be used to interpret the scaling exponent values of urban power laws. If the two correlated entropy maximization processes are in step with each other and in a state of balance, the scaling exponent will approach 1, otherwise, the exponent values will deviate from 1 or even exceed the reasonable range.

## Figures and Tables

**Table 1:** Two types of entropy maximization processes in the evolution of city size distributions.

Entropy Process	Law	Formula	Equation	Complexity
Entropy maximization of frequency distribution	City number law	$N_{m}=N_{1}r_{n}^{m-1}$	(1)	External complexity
Entropy maximization of size distribution	Population size law	$S_{m}=S_{1}r_{s}^{1-m}$	(2)	Internal complexity
	Area size law	$A_{m}=A_{1}r_{a}^{1-m}$	(3)	Internal complexity

**Table 2:** A standard hierarchy with cascade structure based on the pure rank-size distribution of cities (the first four classes and the *M*th class).

Level *m*	Number *N_m_*	Total *T_m_*	Hierarchical Reconstruction of the Rank-Size Distribution (*S_m_* = ln2/2*^m^*^−1^)							
1	1	1	*P*_1_ = 1							
2	2	0.833	*P*_2_ = 1/2				*P*_3_ = 1/3			
3	4	0.760	*P*_4_ = 1/4		*P*_5_ = 1/5		*P*_6_ = 1/6		*P*_7_ = 1/7	
4	8	0.725	*P*_8_ = 1/8	*P*_9_ = 1/9	*P*_10_ = 1/10	*P*_11_ = 1/11	*P*_12_ = 1/12	*P*_13_ = 1/13	*P*_14_ = 1/14	*P*_15_ = 1/15
…	…	…	…	…	…	…	…	…	…	…
*M*	2*^M^*^−1^	ln(2)	1/2*^M^*^−1^	…	…	…	…	…	…	1/(2*^M^*−1)

**Note**: The theoretical foundation was given by Chen [7]. At each level of the hierarchy, the city number is *N_m_*, the total population is *T_m_*, thus the average population size is *S_m_* = *T_m_*/*N_m_*. The notion of the average size will be applied to Figure 3, Figure 4, Figure 5, Figure 6 and Figure 7.

**Table 3:** The reconstructed hierarchical systems of natural cities with cascade structure for the U.S.A., Britain, France, and Germany (2010).

Class	America			Britain			France			Germany		
*m*	*N_m_*	*S_m_*	*A_m_*	*N_m_*	*S_m_*	*A_m_*	*N_m_*	*S_m_*	*A_m_*	*N_m_*	*S_m_*	*A_m_*
1	1	290,503.000	1,194,500.000	1	46,299.000	91,938.879	1	62,242.000	133,817.492	1	28,866.000	40,265.780
2	2	213,517.000	783,975.000	2	10,993.500	20,368.164	2	10,877.000	21,812.770	2	25,354.500	36,563.584
3	4	176,132.500	746,975.000	4	5230.250	8434.331	4	6972.250	17,203.731	4	19,394.000	25,766.545
4	8	115,663.500	501,678.125	8	3946.375	6340.649	8	3541.875	9044.170	8	10,758.875	12,169.475
5	16	60,697.125	236,468.750	16	2034.188	2925.685	16	2097.688	5529.493	16	5168.750	6245.420
6	32	31,127.938	134,110.156	32	1059.219	1802.168	32	1179.563	3175.526	32	2528.500	2940.365
7	64	15,077.375	71,724.609	64	530.453	1000.051	64	483.063	1622.074	64	1131.203	1541.896
8	128	7804.250	3,6437.695	128	246.094	457.709	128	220.945	728.457	128	588.867	836.570
9	256	3992.852	19,124.316	256	96.258	204.449	256	105.547	319.176	256	309.762	455.393
10	512	2068.379	10,039.502	512	38.986	77.708	512	44.010	102.194	512	164.701	248.410
11	1024	1072.855	5235.742	*228*	21.311	19.792	*217*	24.249	22.879	1024	82.616	128.013
12	2048	560.370	2922.583							2048	36.726	55.571
13	4096	288.579	1593.188							*1065*	20.488	18.542
14	8192	145.798	903.534									
15	14,922	75.202	530.333									

**Note**: The original city datasets of the U.S.A., Britain (U.K.), France, and Germany is available, and the link is as follow: http://giscience.hig.se/binjiang/scalingdata/. The unit of area (*A_m_*) is “square meter (m^2^)”, and the unit of size (*S_m_*) of European cities is “block” and that of American cities is “junction”. Population size cannot be directly measured for natural cities.

**Table 4:** The allometric scaling exponents and related parameters and statistics of four self-similar hierarchies of U.S., British, French, and German natural cities (2010).

Type	Parameter and Statistic	U.S.A.	Britain	France	Germany
Size distribution	Fractal dimension (*D*)	1.0827	0.9899	0.9907	1.0247
	Standard error (*σ*)	0.0222	0.0438	0.0344	0.0203
	Goodness of fit (*R*^2^)	0.9954	0.9865	0.9916	0.9965
Area distribution	Fractal dimension (*d*)	1.1416	1.0342	1.0228	1.0596
	Standard error (*σ*)	0.0263	0.0415	0.0609	0.0147
	Goodness of fit (*R*^2^)	0.9942	0.9888	0.9758	0.9983
Size-area allometry	Allometric exponent (*b*)	0.9476	0.9571	0.9578	0.9672
	Standard error (*σ*)	0.0063	0.0179	0.0289	0.0124
	Goodness of fit (*R*^2^)	0.9995	0.9976	0.9937	0.9985
Fractal dimension quotient	*D*/*d*	0.9484	0.9571	0.9686	0.9671
Related quantity	City number (*n*)	31,305	1251	1240	5160
	Level number (*M*)	15	11	11	13
	Scaling range	2~14	2~10	2~10	2~12
	Degree of freedom	11	7	7	9

**Note**: For significance level α = 0.01 and degree of freedom *df* = 7, the threshold value of Pearson correlation coefficient is *R*_0.01, 7_ = 0.7977. The minimum correlation coefficient values of the four cases is *R* = 0.9968.

**Table 5:** The longitudinal and transversal allometric scaling relations of cities and the related growth or distribution functions.

Type	Sub-Type	Basic Models	Main Model	Parameters
Longitudinal allometry	Exponential allometry	$\begin{array}{l} S_{t}=S_{0}e^{ut}\\ A_{t}=A_{0}e^{vt} \end{array}$	$A_{t}=aS_{t}^{b}$	$\begin{array}{l} a=A_{0}S_{0}^{-b}\\ b=v/u \end{array}$
	Logistic allometry	$\begin{array}{l} S_{t}=\frac{S_{\max}}{1+(S_{\max}/S_{0}-1)e^{-vt}}\\ A_{t}=\frac{A_{\max}}{1+(A_{\max}/A_{0}-1)e^{-ut}} \end{array}$	$\begin{array}{l} \frac{A_{t}}{A_{\max}-A_{t}}\\ =a{\left(\frac{S_{t}}{S_{\max}-S_{t}}\right)}^{b} \end{array}$	$\begin{array}{l} a=\frac{A_{0}}{A_{\max}-A_{0}}\\ ÷{\left(\frac{S_{0}}{S_{\max}-S_{0}}\right)}^{b}\\ b=v/u \end{array}$
Crosssectional allometry	Power allometry	$\begin{array}{l} S_{k}=S_{1}k^{-q}\\ A_{k}=A_{1}k^{-p} \end{array}$	$A_{k}=aS_{k}^{b}$	$\begin{array}{l} a=A_{1}S_{1}^{-b}\\ b=p/q \end{array}$
Hierarchical allometry	Exponential allometry	$\begin{array}{l} S_{m}=S_{1}r_{s}^{1-m}\\ A_{m}=A_{1}r_{a}^{1-m} \end{array}$	$A_{m}=aS_{m}^{b}$	$\begin{array}{l} a=A_{1}S_{1}^{-b}\\ b=\ln r_{a}/\ln r_{s} \end{array}$
	Power allometry	$\begin{array}{l} S_{m}=S_{1}N_{m}^{-q}\\ A_{m}=A_{1}N_{m}^{-p} \end{array}$	$A_{m}=aS_{m}^{b}$	$\begin{array}{l} a=A_{1}S_{1}^{-b}\\ b=p/q \end{array}$

**Note**: The symbols are as follows: *t*—time; *k*—rank; *m*—level; *S*—(population) size; *A*—urban area; *a*, *b*, *p*, *q*, *u*, *v*, *r*_a_, *r*_p_, *A*_0_, *A*_1_, *A*_max_, *S*_0_, *S*_1_, *S*_max_ are all parameters (proportionality coefficient, scaling exponent, ratio, capacity, etc.).

## Supplementary Materials

The following are available online at http://www.mdpi.com/1099-4300/20/6/432/s1, File S1: Zipf’s law, hierarchy, and power law (XLSX). This MS Excel file is used to show how to transform a Zipf’s rank-size distribution into a self-similar hierarchy. According to the pure Zipf’s distribution, we generate a standard rank-size sequence such as 1, 1/2, 1/3, … Then according to the generalized 2*^n^* rule, we organize the rank-size sequence into a hierarchy with cascade structure. From the results, we can obtain three geometric sequences: the number of cities at the *m*th class, *N_m_*, the total population of cities at the *m*th class, *T_m_*, and thus the average population size at the mth class is *P_m_* = *T_m_* /*N_m_*. The power law relation between *P_m_* and *N_m_* represents one of the hierarchical scaling laws. File S2: Datasets processed for hierarchies of natural cities (XLSX). This MS Excel file is used to show the original datasets on the natural cities of U.S.A., Britain, France, and Germany, and to illustrate how to organize the city size and urban area sequences into hierarchies with cascade structure. Using the results, we can verify the size-number scaling relation, area-number scaling relation, and the size-area allometric scaling relation.
